# Development of Ginkgo (*Ginkgo biloba*) Nut Starch Films Containing Cinnamon (*Cinnamomum zeylanicum*) Leaf Essential Oil

**DOI:** 10.3390/molecules26206114

**Published:** 2021-10-10

**Authors:** Boo-Kyoung Kim, Hae-Se Lee, Hee-Su Yang, Kyung-Bin Song

**Affiliations:** Department of Food Science and Technology, Chungnam National University, Daejeon 34134, Korea; baak924@naver.com (B.-K.K.); dlgotp@naver.com (H.-S.L.); rmfpxk@naver.com (H.-S.Y.)

**Keywords:** antioxidant activity, biodegradable film, gingko nut starch

## Abstract

There have been many studies on the development biodegradable films using starch isolated from various food sources as a substitute for synthetic plastic packaging films. In this study, starch was extracted from ginkgo (*Ginkgo biloba*) nuts, which were mainly discarded and considered an environment hazard. The prepared starch (GBS) was then used for the preparation of antioxidant films by incorporating various amounts of cinnamon (*Cinnamomum zeylanicum*) essential oil (CZEO), which provides antioxidant activity. The prepared GBS films with CZEO were characterized by measuring physical, optical, and thermal properties, along with antioxidant activity (ABTS, DPPH, and FRAP) measurements. With the increasing amount of CZEO, the flexibility and antioxidant activities of the GBS films increased proportionally, whereas the tensile strength of the films decreased. The added CZEO also increased the water vapor permeability of the GBS films, and the microstructure of the GBS films was homogeneous overall. Therefore, the obtained results indicate that the developed GBS films containing CZEO are applicable as antioxidant food packaging.

## 1. Introduction

The use of synthetic plastic packaging materials, which are not easily decomposed and have serious impact on environmental pollution, is expected to reach about 1 billion tons by 2021 [1]. As an alternative, biodegradable packaging materials have been developed to reduce environmental problems after the use of synthetic plastic packaging materials. Biodegradable packaging materials are mainly prepared from natural biopolymers, such as starch, gelatin, pectin, and cellulose [2]. Among them, starch is abundant in natural polymers, and many studies on starch films for food packaging have been conducted [3,4,5]. Moreover, there have been several studies on starch films developed from many different sources, including unconventional starch sources [6,7,8].

Ginkgo (*Ginkgo biloba*) is grown as a street tree due to its strong adaptability to urban conditions and high tolerance to stress in many countries, such as Korea, Poland, Japan, China, and Europe [9]. Male ginkgo trees are recommended as a street tree, but female ginkgo trees can also grow due to difficulty in sex determination and the production of a large amount of ginkgo nuts every year [10].

Ginkgo nuts are collected and used for food in Southeast Asia, including in Korea and China, but they have ginkgotoxin (4’-O-methylpyridoxine), which leads to abdominal pain and clonic convulsions [11]. Thus, despite the health-improving effects of ginkgo nuts, their intake is limited in some countries [12]. In addition, ginkgo nuts are easily crushed and produce a disgusting smell that is generated by butanoic and nucleic acids from the outer seed coat [13]. However, ginkgo nuts are a potential resource for starch owing to their high starch content. Ginkgo nuts consist of 35% carbohydrate, 6% protein, and 2% fat, and the starch content accounts for up to 70% of dry weight [14,15]. Although many studies have been conducted on starch films for food packaging, there has been no study on ginkgo nut starch (GBS) films. Therefore, ginkgo nuts, which are considered waste and an environmental hazard, were chosen as a novel starch film source in this study.

Cinnamon (*Cinnamomum zeylanicum*) essential oil (CZEO) has exhibited antioxidant and antifungal properties [16]. A biodegradable film with antibacterial and antioxidant capabilities was produced by using pectin and cinnamon leaf essential oil [17]. Antioxidant ability was also shown in biodegradable films with chitosan and cinnamon leaf essential oil [18]. The main components of CZEO are eugenol and *trans*-cinnamaldehyde, which have antioxidant activity [19]. In this study, we prepared GBS films containing CZEO to develop a novel starch film with antioxidant activity, and their physicochemical properties were investigated. Overall, the developed GBS films were found to be applicable as an antioxidant packaging film.

## 2. Results and Discussion

### 2.1. Mechanical Properties

The mechanical properties of films are crucial criteria for choosing food packaging material. The optimum concentration for the preparation of GBS films was starch 2.5% (*w*/*v*) and sorbitol (40% of GBS, *w*/*w*) as a plasticizer, based on preliminary experiments. Table 1 shows that the thickness of GBS films had the tendency to increase slightly as the amount of CZEO increased, suggesting that the aggregation of CZEO droplets had little effect on the film thickness [20]. Compared with foxtail millet starch film [21], GBS-C showed higher TS (11.91 MPa) and lower EB (56.81%). The TS and EB of the foxtail millet starch film were 6.78 MPa and 66.26%, respectively. The TS of GBS-0.1 showed no significant difference from that of GBS-C. On the contrary, for the GBS-0.3 and GBS-0.5, TS decreased from 11.91 MPa to 7.38 and 4.89 MPa. This decrease is a common phenomenon owing to the incorporation of essential oils, which causes an increase in the flexibility and a decrease in the rigidity of films [5,20,22]. In contrast with TS, EB tended to increase as the amount of CZEO increased. These results might infer that starch molecules interact with essential oil molecules [5], resulting in an increase in the flexibility of GBS films. It should also be noted that the EB value of the GBS films increased over 100% for GBS-0.3 (103.56%) and GBS-0.5 (115.01%), resulting in EB values that are better than those of other starch films. The EB value was 99.48% for the foxtail millet starch film with 1.0% clove oil [21] and 26.50% for the corn and wheat starch film with the same concentration of lemon essential oil [20]. These results indicate that GBS–CZEO film can be a biodegradable film material with good flexibility. YM refers to the modulus of elasticity, and the higher the YM value, the higher the stiffness of films [23]. The YM of GBS films decreased with the addition of CZEO in the films, suggesting that the addition of CZEO increased flexibility.

WVP is an important parameter of a film’s physical properties [19]. Measurement of WVP can be helpful in understanding the intermolecular interactions in biodegradable films [24]. In this study, the WVP of GBS films increased as CZEO content increased, except for the films of GBS-0.3 and GBS-0.5, where there was no significant difference. The obtained WVP value of GBS-0.5 was the highest (2.85 × 10^−9^ g/m s Pa) and that of GBS-C was the lowest (2.18 × 10^−9^ g/m s Pa). In general, WVP tends to decrease due to increased hydrophobicity as the amount of added essential oil (EO) increases [25]. However, in the case of GBS films, WVP tended to increase as CZEO was added to the film. It has been known that water vapor transfer is affected by the microstructure of films as well as by the balance of hydrophilicity and hydrophobicity [26]. The increase in WVP is probably owing to the micropores formed during film manufacturing, especially during the drying progress [5]. It also could be due to the loosened film matrix by the addition of CZEO, which showed the decreased TS of the films, where water molecules were transferred more easily.

### 2.2. Optical Properties and Microstructure

Optical properties of films are important characteristics that influence consumer preference. Table 2 shows that the GBS-C was transparent and an almost colorless very light yellow. The addition of CZEO resulted in decreasing L and a values of the films, whereas b value increased from 1.95 ± 0.02 to 2.40 ± 0.05 for GBS-0.5, exhibiting more yellow color. In addition, the total color difference (ΔE) of the GBS films increased according to the various amounts of CZEO. Because phenolic compounds present in CZEO have photosorption, the color change in the films containing CZEO might occur [27]. Moreover, the reason for the increased yellowness of the films was due to the color of eugenol, the major component of CZEO [28]. Similarly, cassava starch films containing cinnamon essential oil became more yellow due to incorporation of coloring components in the essential oil [29].

Low opacity of films makes the appearance of food distinctly visible. When CZEO content increased, the opacity decreased, and the GBS-0.5 (0.15 ± 0.02 Abs/mm) was the most transparent. Similarly, it has been reported that droplets of essential oils penetrate into starch molecules, prevent the generation of the film matrix, and form open structure, resulting in decreased opacity [30]. Therefore, molecular interactions between essential oil and water molecules affect the refractive index of a film, resulting in a change in its opacity [31].

**Figure 1:**
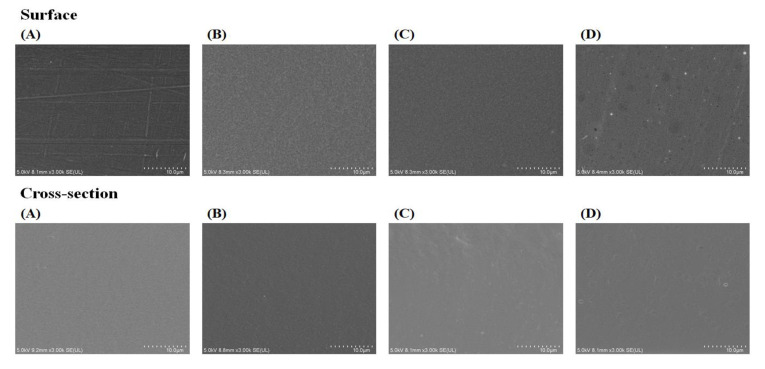
SEM images of GBS films containing CZEO. (**A**) GBS-C; (**B**) GBS-0.1; (**C**) GBS-0.3; (**D**) GBS-0.5.

The microstructure of biodegradable films can be identified through SEM (Figure 1). The surface image suggests that the GBS-C film exhibited smooth structure and had no holes, compared with other starch films. On the contrary, Homayouni et al. [32] reported that tapioca starch film had heterogeneous structure, and Go and Song [33] reported that rye starch film showed cracks on the film surface. In addition, the overall microstructure of GBS films containing CZEO were homogeneous and uniform, except the GBS-0.5 film with few micropores. Although essential oil seems to be dispersed well in the process of film preparation, some extra oil droplets could move to the surface during the drying of films [20]. Thus, some CZEO in the film formulation could evaporate, resulting in holes being made [34]. Similarly, Song et al. [20] reported that the control films of corn and wheat starch showed a smooth surface, but an uneven surface appeared as the amount of added essential oil increased. In the cross-section images, as the content of CZEO increased, slight non-uniformity of the films was shown. Job’s tears starch films with essential oil also showed similar results due to increasing hydrophobic droplets during the drying of films [5]. In addition, as the amount of emulsifier increased according to the concentration of CZEO, Tween 80 molecules could move to the surface of films, affecting the microstructure of the films [20].

### 2.3. FTIR of GBS Films

**Figure 2:**
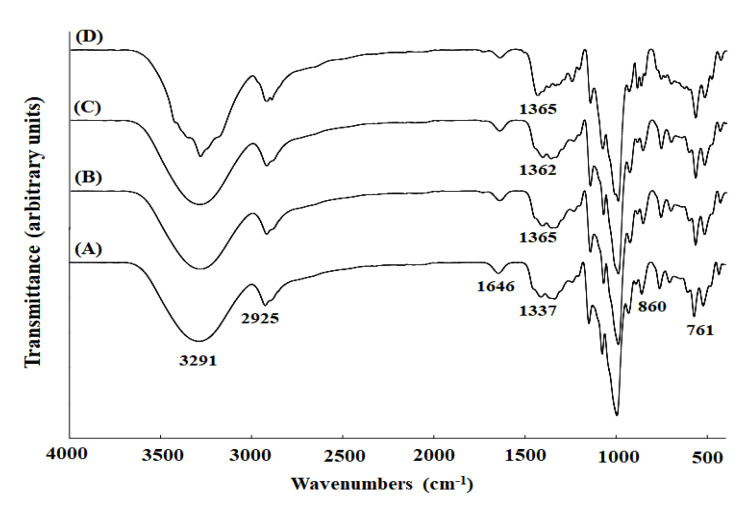
FT-IR spectra of GBS films containing CZEO. (**A**) GBS-C, (**B**) GBS-0.1, (**C**) GBS-0.3, (**D**) GBS-0.5.

FTIR analysis is a technique for identifying the molecular structure and functional groups of films using infrared light. Spectra of the GBS films showed a wide peak at the wavenumber of 3291 cm^−1^ due to the hydroxyl group of ginkgo starch molecules, regardless of the CZEO content [14] (Figure 2). The peak at 3291 cm^−1^ of the CZEO-added film originates from the O-H stretching vibration of starch, plasticizer, and phenolic compounds of essential oil [35]. With the addition of CZEO, a clear shift of peaks was not observed, but the degree of intensity of the films increased. The increased peak amplitude suggests strong hydrogen bonding in the network of CZEO and starch molecules [29]. Moreover, the spectra showed noticeable peaks at 2925 and 1646 cm^−1^. Theses peaks show C-H stretching vibration and strongly bound water of starch molecules [36]. Additionally, there was a band associated with CH_2_ deformation or C-O-H bending of the GBS films at 1337 cm^−1^ [37]. In particular, the higher the CZEO content, the higher the wavenumber shifted (1337 to 1362, 1365cm^−1^). Furthermore, peaks at 860 and 761 cm^−1^ indicate several bonds in the starch linkage, including α-1,4-glycosidic bonds [38].

### 2.4. TGA of GBS Films

**Figure 3:**
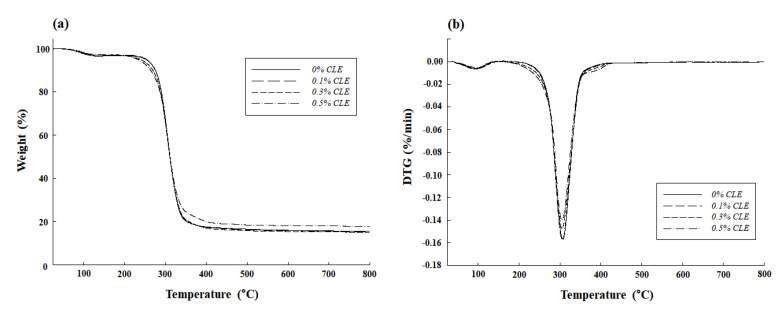
Thermal stability of GBS films containing CZEO. (**a**) TGA, (**b**) DTGA.

Mass loss on thermal decomposition of GBS films was shown in two stages (Figure 3). The first mass loss occurred around 100 °C. This was due to vaporization of water and volatile compounds [35,39]. The second mass loss was the largest and most rapid mass reduction at around the 320 °C region. This was due to the breakdown of the film components, such as starch and plasticizer, and the destruction of hydrogen bonds and starch carbon skeletons [33]. Similarly, two stage weight loss related to the breakdown of starch film was reported in the corn starch-based polymer with added additive [40]. In this study, pyrolysis temperature of GBS-0.1 (319.55 °C), GBS-0.3 (318.58 °C), and GBS-0.5 (318.81 °C) was lower than that of GBS-C (320.57 °C), suggesting that the addition of CZEO decreased the thermal stability of GBS films. In general, all organic compounds were pyrolyzed, and eventually ash only remained. As the concentration of EO increased, the amount of residue of GBS-0.5 increased from 15.05% to 17.80%, compared with GBS-C. An increasing tendency of the residual content of the films seemed to be due to the increase in the amount of aromatic rings, mainly from essential oils [41].

### 2.5. Antioxidant Activities

The antioxidant ability of packaging materials protects foods from reduced shelf life by preventing lipid oxidation, a major reason for deterioration of foods. In this study, several experiments were conducted to examine the antioxidant capacity of the GBS films. Table 3 shows that the antioxidant activity of GBS films increased with CZEO content for three analyses (ABTS radical, DPPH radical, and FRAP). In particular, the GBS-0.5 showed the highest activity, 98.46 ± 0.57%, 58.90 ± 0.74%, and 12.89 ± 0.20 mM FeSO_4_/g film, respectively, whereas the GBS-C showed the lowest. The control film without CZEO had little antioxidant activity, due to polyphenolic compounds in GBS, which were not eliminated from ginkgo nuts [42,43]. In addition, as the content of CZEO increased from 0.1% to 0.3%, the antioxidant activity increased rapidly by more than 5 times. This increase could be attributed to the structure of the phenolic compounds present in CZEO. The content of eugenol present in CZEO was confirmed to be 87.3% using GC-MS [28]. Eugenol, a major compound in CZEO is a powerful phenolic antioxidant [44]. The hydroxyl groups of eugenol react with free radicals and form resonance-stabilized phenolic radicals [45]. Similarly, an effect of phenolic compounds in oregano essential oil on the antioxidant activity of cassava starch film was reported [46].

## 3. Materials and Methods

### 3.1. Materials

Ginkgo nuts were purchased at a local market in Daejeon, Korea. Cinnamon (*Cinnamomum zeylanicum*) leaf essential oil was obtained from Gooworl Co. (Daegu, Korea). Sorbitol and Tween 80 were purchased from Sigma-Aldrich (St. Louis, MO, USA).

### 3.2. Isolation of GBS

GBS was extracted according to the method of Miao et al. [14] and Zheng et al. [47], with minor modifications. After peeling and cleaning ginkgo nuts, the ginkgo nuts were ground with 0.3% NaOH (1:5, *w*/*v*) in a blender and stirred at 4 °C for 24 h. The slurry was strained using a 200 mesh sieve and centrifuged at 3000× *g* to separate starch. A green layer, which remained in the starch, was carefully removed using a spatula. Using distilled water, the starch was dispersed several times and washed to increase the purity of the GBS. The starch solution was then neutralized to pH 7 using 1 N HCI. The solution was then centrifuged at 3000× *g*, washed with distilled water, dried, and shifted into a 200 mesh strainer. The obtained powder was kept at 4 °C. The content of amylose in the obtained GBS was determined to be approximately 22.02 ± 1.95% by the method of Lu et al. [48].

### 3.3. Manufacturing GBS Films

Based on preliminary experiments, the concentrations of GBS, sorbitol, and CZEO were determined. The film-forming solution was formed by stirring GBS (1.25 g), sorbitol (0.5 g), and distilled water (50 mL) at 90 °C for 20 min. The solution was then cooled down with cold water until 70 °C. In contrast with the control film (no CZEO), CZEO (0.05, 0.15, and 0.25 g) and Tween 80 (0.0125, 0.0375, and 0.0625 g) were added to the film-forming solution for the preparation of GBS film with CZEO. After homogenization of the solution at 8000 rpm for 3 min, sonication was performed twice for 5 min each, with a break of 1 min. After filtration with three layers of gauze, 30 mL of the film-forming solution was poured onto a glass plate (10 cm × 13 cm) and dried at constant environment at 25 °C for 15 h. In this study, GBS-C, GBS-0.1, GBS-0.3, and GBS-0.5 indicate the films with 0, 0.1, 0.3, and 0.5% of CZEO, respectively. The prepared films were kept under constant condition (25 °C, RH 50%) for 24 h prior to the experiment.

### 3.4. Characterizations of GBS Films

#### 3.4.1. Mechanical Properties

The GBS films were cut into 2.54 cm × 10 cm. The thickness of the films was gauged at different locations five times with a Mitutoyo 2046-08 micrometer (Tokyo, Japan). Tensile strength (TS), elongation at break (EB), and Young’s modulus (YM) were investigated using an Instron universal M250-2.5 CT testing machine (Testometric Co., Lancashire, UK).

#### 3.4.2. Water Vapor Properties

The films were prepared by cutting them into 2 cm × 2 cm. Water vapor permeability was estimated using a modified cup method [49]. The GBS films were sealed across the top of a polymethylacrylate cup filled with 15 mL of distilled water, stored in a steady environment room (25 °C, 50% RH), and the WVP was calculated by measuring the weight of distilled water permeating through the films every hour.

#### 3.4.3. Optical Properties

Optical properties of the GBS films were measured as described by Kang and Song [5]. L*, a*, and b* are numerical parameters of brightness, redness, and yellowness, respectively. ΔE value represents the difference in the color. Measurements were made on a standard plate (L* = 97.19, a* = −0.34, and b* = 2.35). Color values were obtained using a CR-400 colorimeter (Minolta, Tokyo, Japan). Opacity (Abs/nm) was obtained by dividing the optical density recorded with a Shimadzu spectrophotometer (Kyoto, Japan) at 600 nm by the thickness of the films.

### 3.5. SEM

A field mission scanning electronic microscope (SU8230, Hitachi Co., Tokyo, Japan) was used to evaluate the microstructure of the films. GBS films were stuck onto carbon tape, and platinum coating was performed for 1 min as a pretreatment process. For a cross-sectional image, GBS films were cryo-fractured with liquid nitrogen and mounted perpendicular to the bar. Scanned images of the GBS films were measured at accelerated voltages of 5.0 kV with 3000 magnifications for both surface and cross-section.

### 3.6. Fourier Transform Infrared Spectroscopy (FTIR)

FTIR spectra were obtained using an infrared spectrometer (Vertex 80v, Bruker Optics, Billerica, MA, USA). They were the average of 16 scans in the wavenumber of 4000– 400 cm^−1^.

### 3.7. Thermogravimetric Analysis (TGA)

TGA (TGA/DSC1, Mettler-Toledo, Columbus, OH, USA), which demonstrates the thermal stability of films, was carried out. GBS films (3 mg) with different concentrations of CZEO (0, 0.1, 0.3, and 0.5%) were taken and heated by increasing 10 °C per min from 25 °C to 800 °C.

### 3.8. Antioxidant Activities

Prior to examining the antioxidant activity of GBS films, the following method was carried out for the preparation of film samples: GBS film (0.1 g) was dissolved in 10 mL of water and was shaken at 37 °C for 1 h.

#### 3.8.1. ABTS Radical Scavenging

The sample solution was made up of 2.94 mL of ABTS radical solution and 60 μL of dissolved film sample, as described by Guo et al. [50]. It was voltexed and left in the dark for 10 min. A spectrophotometer was used to obtain the absorbance at 734 nm.

#### 3.8.2. DPPH Radical Scavenging

This analysis was conducted with reference to Lee et al. [49]. Dissolved film sample (0.1 mL) and 3.9 mL of 0.1 mM DPPH radical solution were mixed. The solution was reacted in the dark for 1 h, and the absorbance was measured at 517 nm.

#### 3.8.3. Ferric Reducing Antioxidant Power (FRAP)

This assay was performed with a slight modification of the previously reported method [51]. The FRAP solution was made up of 300 mM acetate buffer (pH 3.6), 20 mM FeCl_3_, and 10 mM TPTZ solution. Dissolved film sample (0.15 mL) and 2.85 mL of FRAP solution were mixed. Then, it was left in the dark for 30 min. The absorbance at 593 nm was measured. Standard curve of ferrous sulfate was used to analyze the ferric reducing ability of the films. The FRAP value is expressed in mM FeSO_4_/g film.

### 3.9. Statistical Analysis

All experimental data were evaluated with Duncan’s multiple range test (*P* < 0.05) to assess significant differences using the SAS program (SAS Institute Inc., Cary, NC, USA). The results were recorded as mean ± standard deviation (SD). All experiments were repeated at least 4 times.

## 4. Conclusions

Through this study, we developed an alternative food packaging material by using starch extracted from ginkgo nuts. Antioxidative GBS films containing various amounts of CZEO were prepared, and their physicochemical properties were evaluated. As the amount of CZEO increased, the GBS films showed higher EB and WVP, but lower TS compared with the control film. In particular, the GBS film containing 0.5% CZEO had the highest EB (115%). The addition of CZEO also improved the antioxidative activity of GBS films. Therefore, these results clearly indicate that the GBS–CZEO films might be utilized as an active packaging material that maintains food quality during storage.

## Figures and Tables

**Table 1:** Physical properties of GBS films containing CZEO.

CZEO (%)	Thickness (mm)	TS (MPa)	EB (%)	Young’s Modulus (MPa)	WVP (10^−9^ g /m s Pa)
0	0.056 ± 0.005 ^a^	11.91 ± 0.94 ^a^	56.81 ± 2.97 ^d^	29.58 ± 5.90 ^a^	2.18 ± 0.13 ^c^
0.1	0.056 ± 0.005 ^a^	10.66 ± 0.96 ^a^	73.67 ± 2.39 ^c^	20.21 ± 1.93 ^b^	2.66 ± 0.05 ^b^
0.3	0.061 ± 0.004 ^a^	7.38 ± 0.97 ^b^	103.56 ± 1.18 ^b^	10.87 ± 1.97 ^c^	2.84 ± 0.05 ^a^
0.5	0.062 ± 0.004 ^a^	4.89 ± 0.71 ^c^	115.01 ± 3.95 ^a^	6.63 ± 1.52 ^c^	2.85 ± 0.10 ^a^

Mean ± SD, *n* = 4. ^a–d^ Any means in the same column followed by different superscripts differ significantly (*p* < 0.05) by Duncan’s multiple range test.

**Table 2:** Optical properties of GBS films containing CZEO.

CZEO (%)	L*	a*	b*	*Δ*E	Opacity (Abs/mm)
0	96.76 ± 0.02 ^a^	−0.34 ± 0.03 ^a^	1.95 ± 0.02 ^c^	-	0.28 ± 0.01 ^a^
0.1	96.45 ± 0.12 ^b^	−0.41 ± 0.04 ^b^	2.25 ± 0.05 ^b^	0.37 ± 0.11 ^b^	0.26 ± 0.02 ^b^
0.3	96.04 ± 0.08 ^c^	−0.43 ± 0.05 ^b^	2.39 ± 0.06 ^a^	0.80 ± 0.08 ^a^	0.20 ± 0.02 ^c^
0.5	96.00 ± 0.06 ^c^	−0.49 ± 0.03 ^c^	2.40 ± 0.05 ^a^	0.84 ± 0.06 ^a^	0.15 ± 0.02 ^d^

Mean ± SD, *n* = 5. ^a–d^ Any means in the same column followed by different superscripts differ significantly (*p* < 0.05) by Duncan’s multiple range test.

**Table 3:** Antioxidant activity of GBS films containing CZEO.

CZEO (%)	ABTS Radical Scavenging (%)	DPPH Radical Scavenging (%)	FRAP (mM FeSO_4_/g Film)
0	3.95 ± 0.20 ^d^	1.98 ± 0.20 ^d^	0.04 ± 0.01 ^d^
0.1	16.39 ± 0.57 ^c^	5.22 ± 0.92 ^c^	0.45 ± 0.06 ^c^
0.3	76.16 ± 0.44 ^b^	20.58 ± 0.67 ^b^	4.37 ± 0.07 ^b^
0.5	98.46 ± 0.57 ^a^	58.90 ± 0.74 ^a^	12.89 ± 0.20 ^a^

Mean ± SD, *n* = 5. ^a–d^ Any means in the same column followed by different superscripts differ significantly (*p* < 0.05) by Duncan’s multiple range test.

## Sample Availability

Samples of the compounds are not available from the authors.
